# Visual field preferences of object analysis for grasping with one hand

**DOI:** 10.3389/fnhum.2014.00782

**Published:** 2014-10-01

**Authors:** Ada Le, Matthias Niemeier

**Affiliations:** Psychology, University of Toronto ScarboroughToronto, ON, Canada

**Keywords:** grasping, visual field effect, contralateral, lateralization, unimanual

## Abstract

When we grasp an object using one hand, the opposite hemisphere predominantly guides the motor control of grasp movements (Davare et al., 2007; Rice et al., 2007). However, it is unclear whether visual object analysis for grasp control relies more on inputs (a) from the contralateral than the ipsilateral visual field, (b) from one dominant visual field regardless of the grasping hand, or (c) from both visual fields equally. For bimanual grasping of a single object we have recently demonstrated a visual field preference for the left visual field (Le and Niemeier, 2013a,b), consistent with a general right-hemisphere dominance for sensorimotor control of bimanual grasps (Le et al., 2014). But visual field differences have never been tested for unimanual grasping. Therefore, here we asked right-handed participants to fixate to the left or right of an object and then grasp the object either with their right or left hand using a precision grip. We found that participants grasping with their right hand performed better with objects in the right visual field: maximum grip apertures (MGAs) were more closely matched to the object width and were smaller than for objects in the left visual field. In contrast, when people grasped with their left hand, preferences switched to the left visual field. What is more, MGA scaling with the left hand showed greater visual field differences compared to right-hand grasping. Our data suggest that, visual object analysis for unimanual grasping shows a preference for visual information from the ipsilateral visual field, and that the left hemisphere is better equipped to control grasps in both visual fields.

## Introduction

Vision plays a crucial role in the sensorimotor control of actions. To grasp an object, the brain may analyze visual input to estimate grasp-relevant object features. For example, an object's shape and size, center of mass, and apparent surface friction are relevant to identify grasp points on the surface of the object (Blake, 1992; Voudouris et al., 2012). These points may then guide grasp movements, especially during “precision grips,” such as with the thumb in opposition to the index finger of the same hand (Napier, 1956).

Grasp movements originate from visuomotor control mechanisms that are computed by a cortical network in the inferior frontal and intraparietal cortex (Castiello, 2005; Castiello and Begliomini, 2008; Grafton, 2010; Davare et al., 2011). The hub of this dorsolateral network is the anterior intraparietal sulcus (aIPS; Culham et al., 2003; Frey et al., 2005) which has been shown to implement the initial steps of the visual analysis for grasps (Rizzolatti and Luppino, 2001; Tunik et al., 2005, 2008; Culham and Valyear, 2006; Castiello and Begliomini, 2008; Grafton, 2010; Le et al., 2014) as well as perform ensuing transformations for visuomotor control (Castiello, 2005; Davare et al., 2007, 2010; Cavina-Pratesi et al., 2010; Koch et al., 2010; Monaco et al., 2013; Theys et al., 2013).

Disrupting aIPS activity with transcranial magnetic stimulation (TMS) impacts the prehension component of reach-to-grasp trajectories in a contralateral manner such that stimulation in one hemisphere affects the movements of the hand on the respective opposite side of the body (Rice et al., 2007). Nevertheless, TMS paradigms also suggest a left aIPS dominance for certain aspects of the grasp such as grip force control (Davare et al., 2007).

Consistent with a relative left-hemisphere dominance are data from behavioral and fMRI studies. For instance, the scaling of right hand grasps is less affected by size-contrast illusions than grasps with the left hand (Gonzalez et al., 2006). Also, grip-type selection predominantly activates the left ventral premotor cortex, regardless of the hand dominance (Martin et al., 2011). In addition, other fine motor skills show an equivalent right hand advantage (Serrien et al., 2006). In sum, sensorimotor control of grasping with one hand shows a contralateral organization with a relative dominance of the left hemisphere on the side of motor output.

To date, however, it is unclear whether lateralized motor control is complemented by an equivalent visual field preference or dominance at an earlier stage of visual object analysis for grasp computations. That is, does grasp control (a) rely more on visual object analysis in the contralateral than the ipsilateral visual field, does control (b) show a general preference for the right visual field, or does it (c) use information from both visual fields equally?

To our knowledge, data about visual field preferences for grasping are largely incomplete. Le and Niemeier (2013a,b) tested bimanual rather than unimanual grasping and found a visual field preference for the left visual field. This shows that it is feasible to assume that unimanual grasping prefers one visual field as well, given that the mechanisms underlying bimanual grasping and unimanual grasping partially overlap (Le et al., 2014). Even so, this does not necessarily imply that unimanual grasping would show a visual field preference. Shmuelof and Zohary (2006) found that brain activity in the right hemisphere varied as a function of visual field but not left hemisphere activity. However, these authors had participants observe unimanual grasp actions, rather than perform them; thus, their observations can only provide indirect evidence about visual object analysis for grasping.

To directly answer the question of a visual field preference for unimanual grasping, here we used a visual field paradigm. Participants grasped either with their right hand (Experiment 1) or left hand (Experiment 2) while viewing objects either in their left or right visual field. For right-hand grasping, we found that maximum grip apertures (MGA) were more closely matched to object width and were smaller for objects in the right visual field than for objects in the left visual field, indicating a left hemisphere advantage. In contrast, for left-hand grasping, we found a left visual field advantage, consistent with a greater involvement of the right hemisphere. What is more, left-hand grasping showed greater differences between left and right visual field. Together, our data suggest that visual object analysis for unimanual grasping prefers visual information from the contralateral visual fields and that the left hemisphere may be better equipped to control right-handed grasp movements in both visual fields.

## Methods

### Participants

A total of 49 healthy undergraduate students (Experiment 1: *N* = 28, 14 females, mean age of 20 years; Experiment 2, *N* = 21, 12 females, mean age of 21 years) gave their informed and written consent to participate in this study. All participants had normal or corrected to normal vision, and were right handed as confirmed with the Edinburgh handedness inventory [Experiment 1: laterality quotient = 76.4; Experiment 2: laterality quotient = 92.2; *t*_(47)_ = −2.36, *p* = 0.023; Oldfield, 1971]. All procedures were approved by the Human Participants Review Sub-Committee of the University of Toronto and therefore have been performed in accordance with the ethical standards laid down in the 1964 Declaration of Helsinki.

### Apparatus

Participants sat in a dark room (*L_v_* = 0.01 cd/m^2^, measured at the location of the object) at a table with their head stabilized in a chin rest and their sight controlled by a set of Plato goggles (Translucent Technology, Toronto). A 19 inch LCD monitor (1024 × 768 pixels, 100 Hz refresh rate) was mounted on the table 60 cm away from the participant at eye level. Twenty centimeters in front of the monitor and aligned with the participant's body midline, we installed a pedestal on which we placed a gray wooden block (75 mm by 50 mm by 24 mm) 3 cm below eye level. Also, a tactile marker on the table 24 cm in front of the participant's trunk served as a start position for the hand movements. Hand trajectories were recorded with three infrared Qualisys motion tracking cameras (Qualisys, 240 Hz) and passive spherical markers (10 mm in diameter) fixed on the tips of the index finger, thumb, and on the wrist at the junction of the ulna and the carpal. Eye position of the left eye was tracked while the Plato goggles were transparent using an EyeLink II system (SR Research, Ottawa; sampling rate: 250 Hz) to allow us to exclude trials with improper fixation. Both, eye tracking and visual stimuli were controlled by Matlab (MathWorks) together with the Psychophysics Toolbox (Brainard, 1997; Pelli, 1997) and Eyelink Toolbox extensions (Cornelissen et al., 2002). The Plato goggles were controlled by a custom-made program.

### Procedure

**Figure 1:**
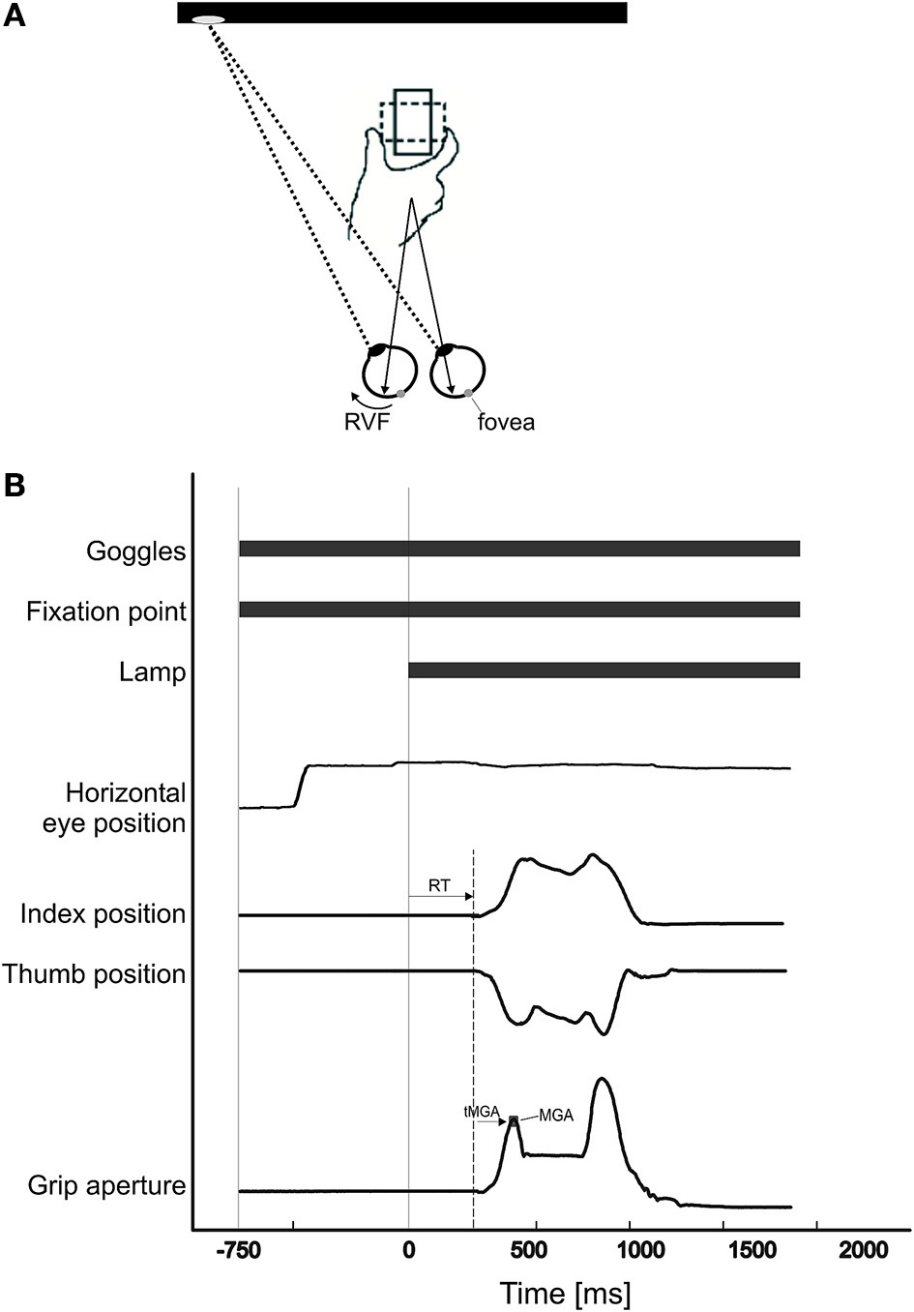
**(A) Participants were asked to fixate either to the left or right of the object**. When they fixated to the left, the object was in their right visual field. When they fixated to the right, the object was in their left visual field. The object was presented either horizontally (dotted-line; i.e., participants grasp the widest dimension of the object), or vertically (solid-line; i.e., participants grasp the narrow dimension of the object). To grasp the object, participants used the index finger and thumb to pick up the object from its left and right sides. **(B)** Time sequence of one trial: As soon as the Plato goggles opened, the fixation point (FP) was visible and participants fixated on it. Seven hundred and fifty milliseconds after the onset of the FP, the monitor (“lamp”) turned white, thus illuminating the object. As soon as the “lamp” came on, participants grasped the object. Reaction time was calculated as the time from “lamp” onset to movement onset. Time of maximum grip aperture (tMGA) was calculated as the time from movement onset to maximum grip aperture (MGA).

A spatial and temporal illustration of an experimental trial is given in Figure 1 and adopted from Le and Niemeier (2013a). In brief, at the beginning of each trial, the Plato goggles were opaque and the participants rested their index finger and thumb adjacent to each other on the tactile start position. Meanwhile, the experimenter placed, in darkness, the object on the pedestal so that its 75 × 50 mm side faced the participant. Its orientation was chosen to be horizontal or vertical following a random protocol generated by the Matlab program and displayed as a small-fonted “H” or “V” on the monitor (i.e., invisible through the translucent goggles). Next, hand position recordings were started and this triggered the goggles to turn transparent so that the pupils became visible to the eye tracker. The Matlab program waiting for this signal then presented a red fixation dot (~0.5 visual degrees in diameter) 15 visual degrees to the left or to the right of the object. Participants were asked to move their eyes to the dot and fixate it to manipulate visual field presentation. Note that fixating to the left of the object brings the object into the right visual field (“right VF”), whereas fixating to the right brings the object into the left visual field (“left VF”). Also, it is important to note that the low luminance levels during the initial fixation period made it very unlikely that any object information, useful for grasping, entered the visual system (e.g., in pilot tests no conscious object perception was possible even with 20 min dark adaptation, whereas the actual experiment prevented dark adaptation). Seven hundred milliseconds after initial fixation, the screen background became white, thus illuminating the object so that it appeared in the participant's right or left visual field (manipulating visual fields with different fixation locations is one commonly used strategy, e.g., Macaluso et al., 2002; it avoids biomechanical confounds because reach-to-grasp movements are kept the same). Participants then moved their hands (Experiment 1: right hand; Experiment 2: left hand) to grasp the object at its left and right sides and lift it off the pedestal (i.e., a horizontal object orientation required grasps across the wide object side, a vertical orientation required grasps across the narrow side). Only precision grasps with index finger and thumb were permitted, power grasps were not permitted because it is unclear to which extent they require detailed grasp point computations (Ehrsson et al., 2000). Participants' grasps were visually monitored during the illumination period to ensure they grasped as instructed. Participants were told to move as fast as possible without sacrificing accuracy. After 2500 ms, the hand tracking stopped, the monitor turned black, and the goggles became opaque once again. Thirty of such trials were conducted in each block, and there were 2 blocks in total.

### Data analysis

Hand tracking data were preprocessed trial by trial with the Qualisys software and then further analyzed together with the eye position data in MATLAB. Initiation and termination of hand movement for each trial was determined based on a 5% criterion of peak velocity of that trial (right-hand grasping: thumb *M* = 68.8 mm/s, *SD* = 25.7, index *M* = 52.3 mm/s, *SD* = 9.7; left-hand grasping: thumb *M* = 64.7 mm/s, *SD* = 7.8, index *M* = 54.5 mm/s, *SD* = 3.5; movement start and end identification was further monitored on a trial-by-trial basis, and any inaccuracies were manually corrected). The MGA was defined as the largest distance between the index finger and thumb during our participants' reach-to-grasp movements. The data were visually inspected to identify and exclude invalid trials (20% were invalid trials for right-hand grasping, and 16% for left hand grasping). Exclusion criteria were: eye fixation errors after the onset of the white screen (e.g., not maintaining fixation throughout the trial by deviating more than 3.75 visual degrees away from fixation; see Le and Niemeier, 2013a), reaction times shorter than 50 ms, and incomplete or noisy hand trajectories due to artifacts.

For each individual participant, we then extracted seven dependent variables, mostly in line with our previous visual field parameters (Le and Niemeier, 2013a,b). The first four variables were measures of grasp movement metrics: To look at MGA scaling, we calculated slopes ([MGA of wide object width – MGA of narrow object width]/[wide object width – narrow object width]), which has traditionally been used to indicate grasp proficiency (see Smeets and Brenner, 1999 for a review). A slope of 0 indicates no scaling of the MGA to the object, whereas a slope of 1 indicates perfect scaling, and so higher slopes reflect greater proficiency in grasping, although the typical range is 0.7–1 (Smeets and Brenner, 1999). The second and third measure were the absolute size of MGA and MGA in proportion to the respective object width (proportional MGA = absolute MGA/object width; calculated for narrow and wide widths separately). Here, smaller values closer to the actual width of the respective object reflect greater grasp proficiency (Schlicht and Schrater, 2007; thus proportional MGA values closer to but larger than 1 are more ideal). We examined standard deviations of MGA, calculated for each participant separately, as a fourth measure of grasp metrics to allow for comparisons with our previous studies on bimanual grasping (Le and Niemeier, 2013a,b). In the latter studies we found reduced variability of MGA for the left visual field together with other signs of visual field dominance for that side and a matching right-hemisphere dominance of bimanual grasping (Le et al., 2014). Three additional variables inspected the timing of grasp movements: Reaction time captured the time from the object becoming visible to the fingers starting to move, time of MGA (tMGA) measured the time from movement onset to MGA, and total movement time measured the time from movement onset till end of the movement at object contact.

## Results

### Experiment 1: right-hand grasping

**Figure 2:**
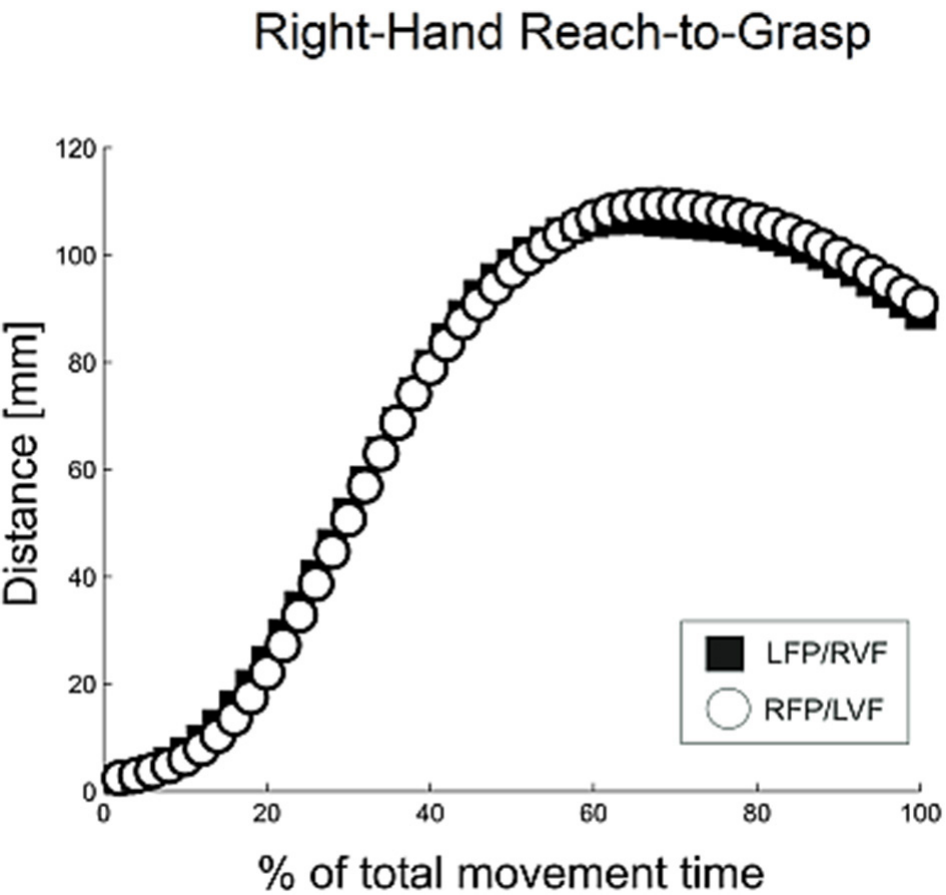
**Distance between digits for right-hand reach-to-grasp, calculated using normalized aperture trajectories**. For normalization, time points were first converted to a percent of the total number of time points, and then movement data were re-sampled using a gliding truncated Gaussian-based weighted average to give 100 equal steps. Using these normalized trajectories, the aperture was calculated as the average distance between the thumb and index finger for each time point.

Grasping trajectories showed a MGA during the second half of the movement (~66% of total movement time), which resembled the typical trajectories for unimanual grasping (Figure 2; see Jeannerod, 1984; Tresilian and Stelmach, 1997; Castiello, 2005).

#### Visual field effects on the MGA of reach-to-grasp movements

**Figure 3:**
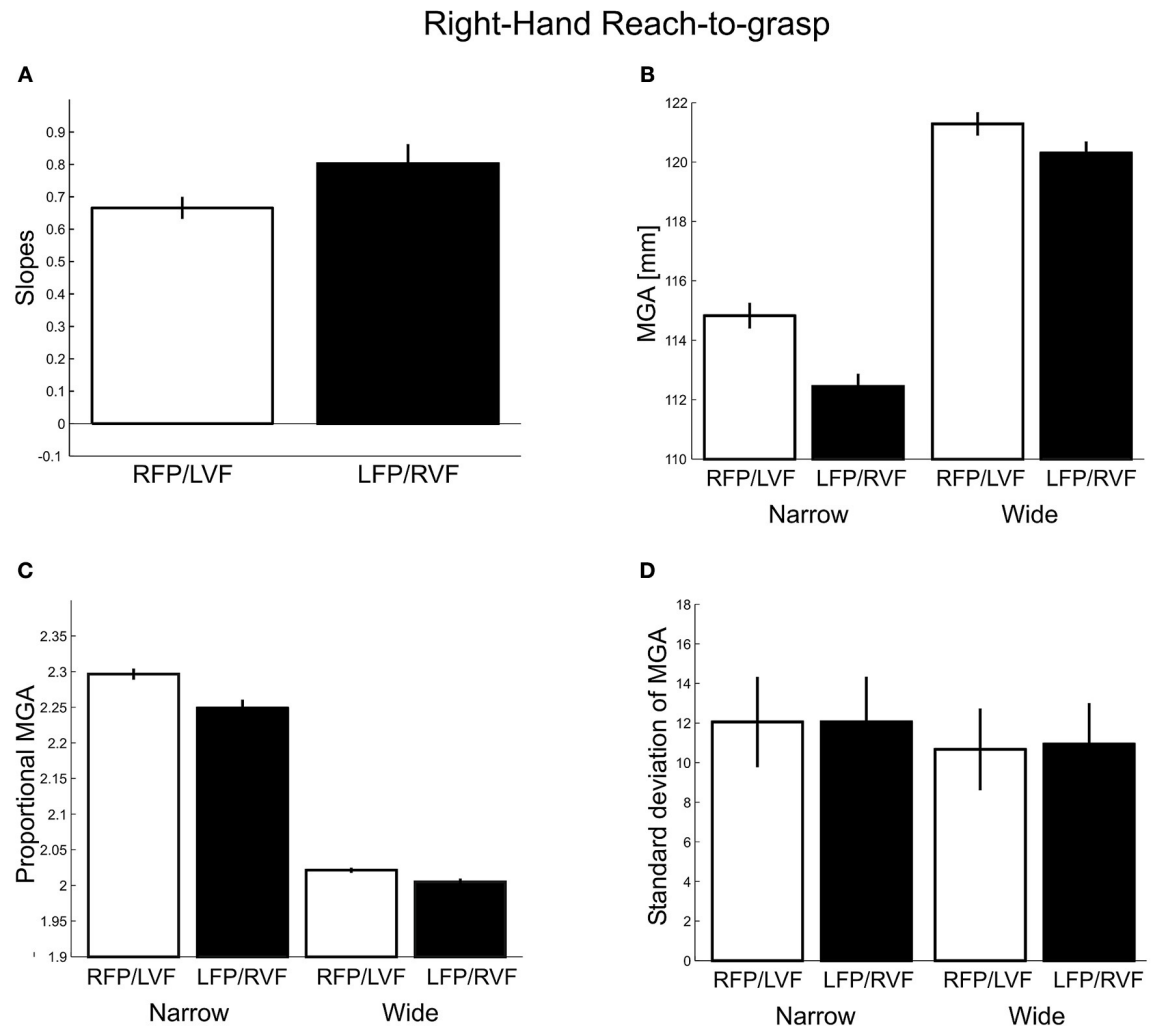
**Right-hand grasping: Measures of maximum grip aperture (MGA). (A)** MGA slopes for different object widths for right fixation/left visual field (RFP/LVF) and left fixation/right visual field (LFP/RVF). Slope = [MGA of wide object width – MGA of narrow object width]/[wide object width – narrow object width]. **(B)** MGA size during grasping for narrow and wide object widths for the RFP/LVF and LFP/RVF. **(C)** Proportional MGA during grasping for narrow and wide object widths for the RFP/LVF and LFP/RVF. Proportional MGA = absolute MGA/object width. **(D)** Standard deviation of MGA during grasping for narrow and wide object widths.

To see whether the metrics of grasping movements were influenced by visual field, we studied four measures related to MGA for the two visual fields separately: MGA scaling, absolute and proportional MGA size, and MGA variability (see Methods). For MGA scaling (Figure 3A), we obtained values of 0.67 and 0.80 for the left VF and right VF, respectively; with the slopes for left VF being slightly below the lower end of the normal range (0.7–1.0, Smeets and Brenner, 1999). This difference was significant [*t*_(27)_ = 3.69, *p* = 0.001, *d* = 1.42; 22 out of 28 participants showed the effect], indicating a right visual field advantage [although both slopes were larger than zero, *t*_(27)_ = 24.14, *p* < 0.001; *t*_(27)_ = 13.65, *p* < 0.001, respectively, indicating that both visual fields permitted functional grasps].

Because using slopes alone to capture grasp performance could overlook systematic errors in grasping, next we submitted absolute MGA values (Figure 3B) to a repeated-measures ANOVA with factors “visual field” and “object width.” We observed a main effect of object width [*F*_(1, 27)_ = 260.02, *p* < 0.001, η^2^ = 0.83] and a main effect of visual field [*F*_(1, 27)_ = 116.85, *p* < 0.001, η^2^ = 0.05] such that MGA was larger in the left VF, consistent with reports that MGA increases as grasping becomes more difficult (Schlicht and Schrater, 2007). A significant visual field × object width interaction [*F*_(1, 27)_ = 15.32, *p* < 0.001, η^2 = 0.01]^ reflected that for the narrow object width the MGA was larger in the left VF than the right VF [LVF – RVF = 2.38 mm; *t*_(27)_ = −8.13, *p* < 0.001, *d* = 3.12; all participants showed the effect]. For the wide object width, MGA was similarly modulated by visual field, although somewhat less [LVF – RVF = 0.98 mm; *t*_(27)_ = −5.98, *p* < 0.001, *d* = 2.30; 25 of 28 participants showed the effect]. The reduced visual field effect could be due to the restrictions of the hand span on the size of MGA for wider objects, thus, the interaction could reflect a ceiling effect. Consistent with this, we found that skewness values of MGA for individual participants were significantly more negative for the wide object size [*F*_(1, 24)_ = 7.52, *p* = 0.01]. Nevertheless, it is interesting to note that we have made the same kind of observation of smaller visual field effects for a larger among a similarly sized set of object sizes for bimanual precision grasps with no apparent biomechanical constraints and ceiling effects (Le and Niemeier, 2013a; also see the MGA analysis for Experiment 2 of the current study).

To further ensure that our approach of inspecting absolute MGA values did not overlook any effects, we submitted proportional MGA (Figure 3C) values to a repeated-measures ANOVA with factors “visual field” (left, right), and “object width” (narrow 50 mm, wide 75 mm), and found a significant main effect of visual field [*F*_(1, 27)_ = 112.17, *p* < 0.001, η^2^ = 0.01] and its interaction with object width [*F*_(1, 27)_ = 20.83, *p* < 0.001, η^2^ = 0.003], such that the proportional MGA values were closer to 1 when the object was in the right visual field, and especially so for the narrow object width (narrow: LVF – RVF = 0.05 mm; wide: LVF – RVF = 0.02 mm). Lastly, proportional MGAs were closer to 1 for the wider object width compared to the narrow object width [wide – narrow = −0.25 mm; “object width” factor: *F*_(1, 27)_ = 866.03, *p* < 0.001, η^2^ = 0.94].

As a fourth measure of grasp metrics adopted from our previous studies (Le and Niemeier, 2013a,b): we examined standard deviations of MGA calculated for each participant separately (Figure 3D). However, in contrast to our earlier work we found no main effect of visual field [*F*_(1, 27)_ = 0.51, *p* = 0.48] or interactions with object size [*F*_(1, 27)_ = 1.60, *p* = 0.22]. The narrow object width yielded more MGA variability than the wide object width [Narrow – Wide = 1.25 mm; *F*_(1, 27)_ = 73.70, *p* < 0.001, η^2^ = 0.44; 26 out of 28 participants showed this trend; cf. Ganel et al., 2008 and Heath et al., 2011]. In sum, three out of four grasp metric variables showed a preference for the right visual field.

#### Visual field effects on timing of reach-to-grasp movements

**Figure 4:**
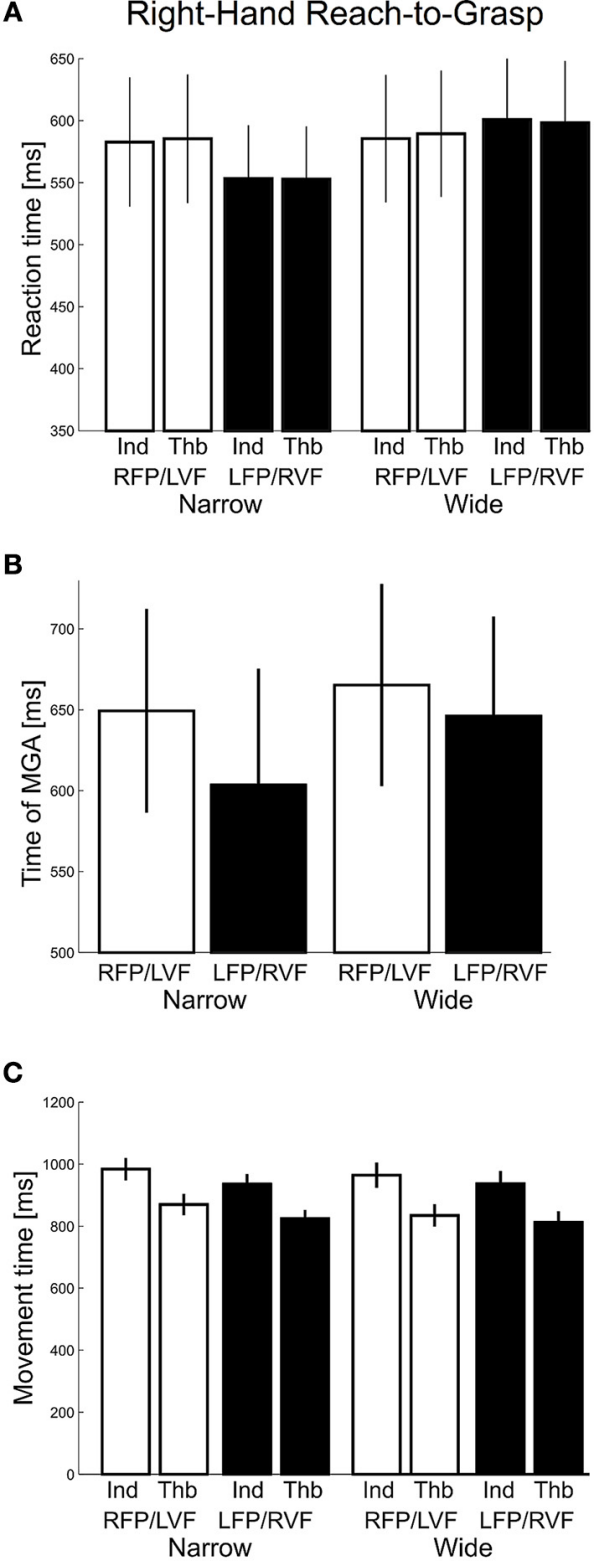
**Right-hand grasping: Temporal measures of hand movements. (A)** Average reaction time for grasping for narrow and wide object width. Thb = thumb, Ind = index. **(B)** Time of maximum grip aperture (MGA) for grasping for narrow and wide object width. **(C)** Total movement time for grasping for narrow and wide object width.

Next, we inspected the temporal aspects of grasping for visual field differences. Reaction times (Figure 4A) were submitted to a repeated-measures ANOVA with factors “visual field” (left, right), “object width” (narrow, wide), and “digit” (thumb, index). We found no main or interaction effects involving visual field, object width, or digit (*F*'s ≤ 2.40, *p*'s ≥ 0.13). The tMGA revealed significantly delayed tMGAs for the wider object width compared to narrow [Wide – Narrow = 15.92 ms, *F*_(1, 27)_ = 14.48, *p* < 0.001], as expected given typical grasp kinematics (see Smeets and Brenner, 1999). However, we did not observe significant main effects of visual field [*F*_(1, 27)_ = 1.51, *p* = 0.23], although there was a non-significant trend for earlier MGA times in the right VF condition, especially for the narrow object width [LVF − RVF = 45.9 ms; Figure 4B; visual field x object width interaction: *F*_(1, 27)_ = 2.81, *p* = 0.11]. Again, this interaction trend is consistent with previous reports of visual field effects modulated by object size (see Le and Niemeier, 2013a). Lastly, for total movement time, main effects of visual field and object size, along with all interaction effects, were not significant (*F*'s ≤ 3.67, *p* ≥ 0.07). We found a main effect of digit [*F*_(1, 27)_ = 50.05, *p* < 0.001, η^2^ = 0.05] such that the thumb arrived at the object before the index finger (Figure 4C), perhaps due to the different biomechanical as well as task/goal constraints (e.g., Melmoth and Grant, 2012). The results here suggest that visual fields did not affect overall timing during grasping with the right hand.

#### Training effects

To look for learning effects we calculated group averages based on the first and second half of grasping trials for each participant. Though this reduced the power of our data, trends in both halves of trials showed the same direction of visual field effects as the original analysis.

### Experiment 2: left-hand grasping

**Figure 5:**
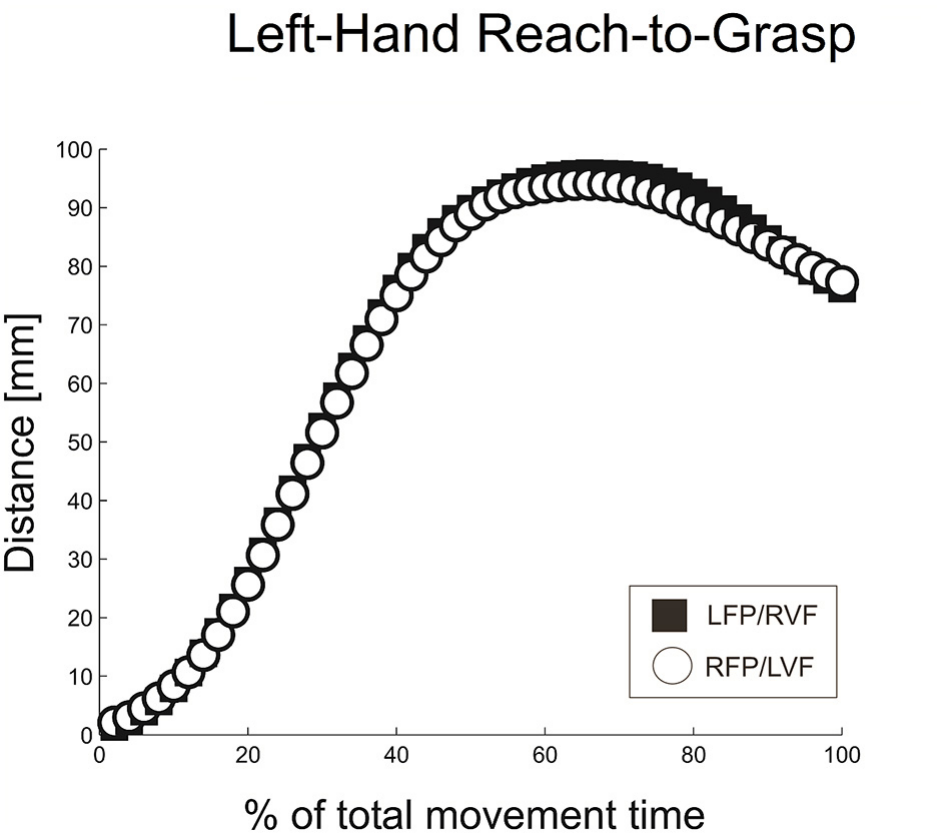
**Distance between digits for left-hand reach-to-grasp, calculated using normalized aperture trajectories**.

Similar to right-hand grasping, the left-hand grasping trajectories showed a MGA during the second half of the movement (~68% of total movement time), which resembled the typical trajectories for unimanual grasping (Figure 5; see Jeannerod, 1984; Tresilian and Stelmach, 1997; Castiello, 2005).

#### Visual field effects on the MGA of reach-to-grasp movements

**Figure 6:**
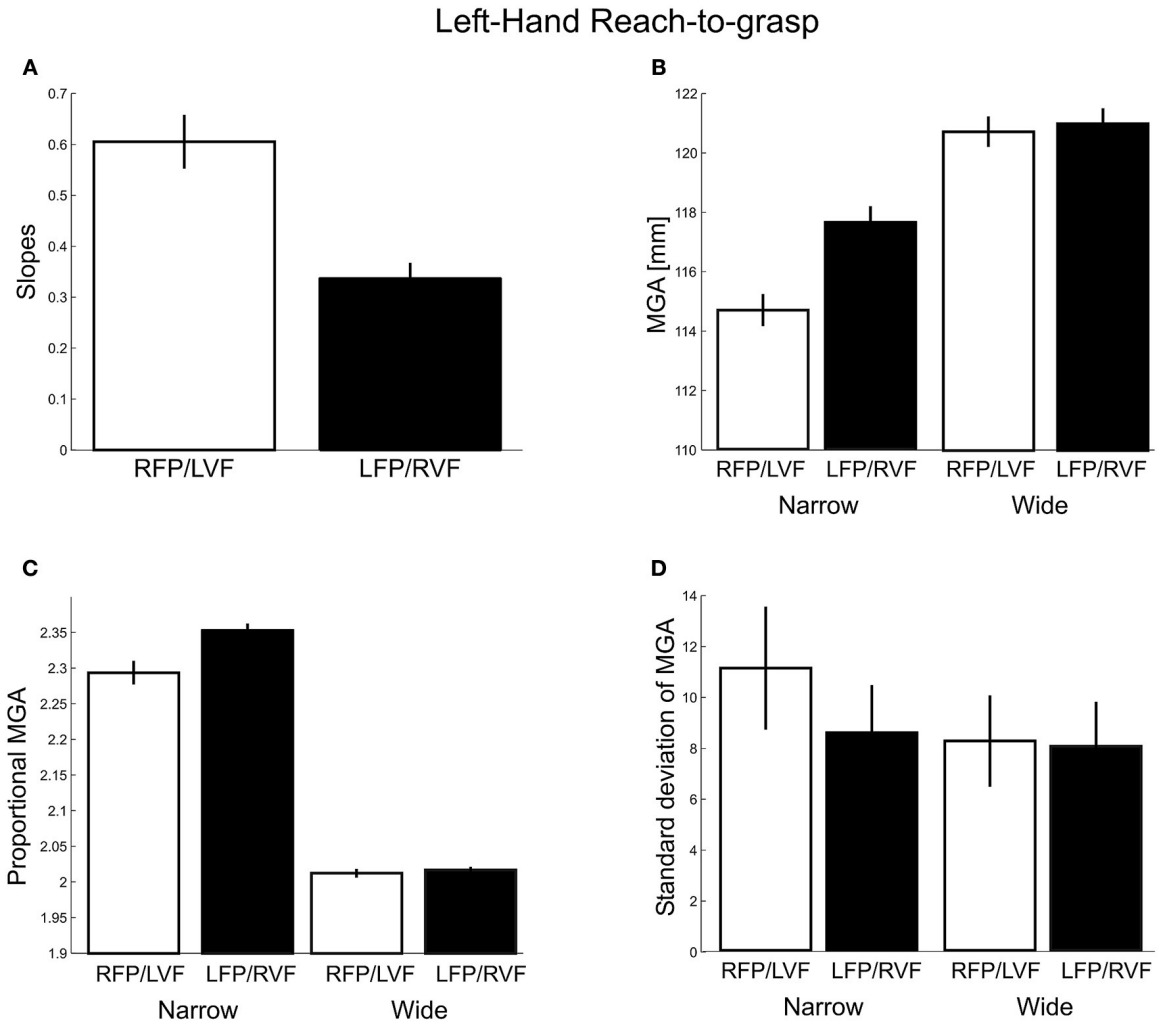
**Left-hand grasping: Measures of maximum grip aperture (MGA). (A)** MGA slopes for different object widths for right fixation/left visual field (RFP/LVF) and left fixation/right visual field (LFP/RVF). **(B)** MGA size during grasping for narrow and wide object widths for the RFP/LVF and LFP/RVF. **(C)** Proportional MGA during grasping for narrow and wide object widths for the RFP/LVF and LFP/RVF. Proportional MGA = absolute MGA/object width. **(D)** Standard deviation of MGA during grasping for narrow and wide object widths.

As for MGA scaling (Figure 6A), we obtained slopes of 0.60 and 0.34 for the left VF and right VF, respectively. This difference was significant [*t*_(20)_ = −7.60, *p* < 0.001, *d* = 3.40; all participants showed the effect], indicating a left visual field advantage [although both slopes were larger than zero, *t*_(20)_ = 11.48, *p* < 0.001; *t*_(20)_ = 10.82, *p* < 0.001, respectively, indicating that both visual fields permitted functional grasps].

Next, absolute MGA values (Figure 6B) submitted to a repeated-measures ANOVA with factors “object width” and “visual field” revealed a main effect of object width [*F*_(1, 20)_ = 141.89, *p* < 0.001, η^2^ = 0.70] and a main effect of visual field [*F*_(1,20)_ = 36.17, *p* < 0.001, η^2^ = 0.08] such that MGA was larger in the right VF. A significant visual field x object width interaction [*F*_(1, 20)_ = 57.82, *p* < 0.001, η^2^ = 0.06] reflected that for the narrow object width the MGA was larger in the right VF than the left VF [RVF – LVF = 2.96 mm; *t*_(20)_ = 7.18, *p* < 0.001, *d* = 3.21; all participants showed the effect]. For the wide object width, MGA was not significantly modulated by visual field [RVF – LVF = 0.27 mm; *t*_(20)_ = 1.42, *p* = 0.17]. To test for possible ceiling effects, an additional analysis of skewness of the MGA data was conducted but found no evidence for more negative skewness for the wider object width [*F*_(1, 19)_ = 0.778, *p* = 0.389].

An ANOVA conducted for proportional MGAs (Figure 6C) revealed a significant main effect of visual field [*F*_(1, 20)_ = 38.48, *p* < 0.001, η^2^ = 0.01] and an interaction with object width [*F*_(1, 20)_ = 58.67, *p* < 0.001, η^2^ = 0.007], such that the proportional MGA values were closer to 1 when the object was in the left visual field, and especially so for the narrow object width (narrow: LVF – RVF = −0.05 mm; wide: LVF – RVF = −0.005 mm). Lastly, proportional MGAs were closer to 1 for the wider object width compared to the narrow object width [wide − narrow = −0.29 mm; *F*_(1, 20)_ = 1270.80, *p* < 0.001, η^2^ = 0.95].

Lastly, we examined the standard deviations of MGA (Figure 6D). Here we found significant main effects of visual field [*F*_(1, 20)_ = 85.58, *p* < 0.001, η^2^ = 0.25] and its interaction with object size [*F*_(1, 20)_ = 200.28, *p* < 0.001, η^2^ = 0.18]. That is, the MGA was less variable in the right VF than in the left VF, especially for the narrow object width (narrow: RVF – LVF = −2.55 mm; wide: RVF – LVF = −0.21 mm; all participants showed this effect). Moreover, the narrow object width yielded more MGA variability than the wide object width [Narrow – Wide = 1.70 mm; *F*_(1, 20)_ = 68.15, *p* < 0.001, η^2^ = 0.38]. These results could suggest greater proficiency in the left compared to the right visual field (in contrast to the three previous measures of left-hand grasping), together with a left-hemisphere dominance for the underlying neural processes. However, inconsistent with this interpretation, right-hand grasping did not produce a comparable right visual field advantage. Given this, we will re-analyze our different dependent measures in factor analyses near the end of the Results. In sum, three out of four grasp metric variables showed a preference for the left visual field, and one showed a preference for the right visual field.

#### Visual field effects on timing of reach-to-grasp movements

**Figure 7:**
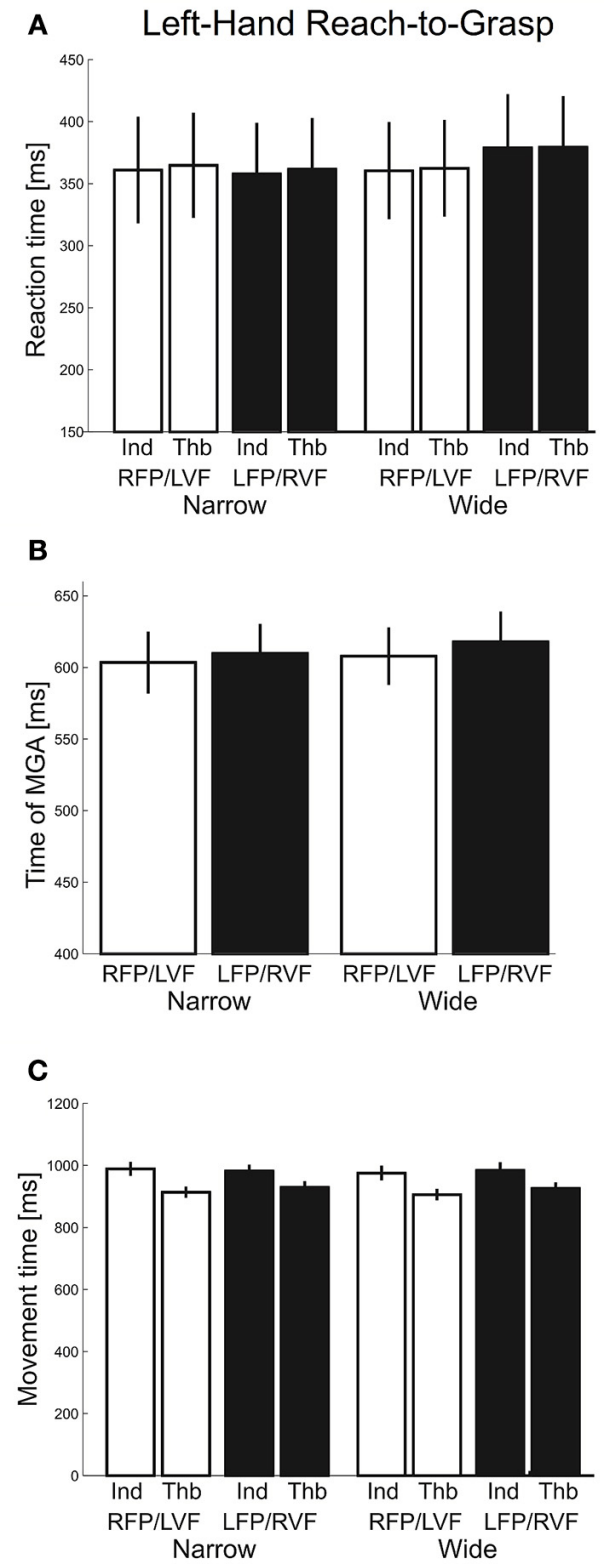
**Left-hand grasping: Temporal measures of hand movements. (A)** Average reaction time for grasping for narrow and wide object width. **(B)** Time of maximum grip aperture (MGA) for grasping for narrow and wide object width. **(C)** Total movement time for grasping for narrow and wide object width.

Next, we inspected reaction times (Figure 7A), which were submitted to a repeated-measures ANOVA with factors “visual field” (left, right), “object width” (narrow, wide), and “digit” (thumb, index). We found no main or interaction effects involving visual field, object width, or digit (*F*'s ≤ 3.45, *p*'s ≥ 0.08). The tMGA showed no significant effects either (Figure 7B. *F*'s ≤ 1.40, *p*'s ≥ 0.25). Lastly, for total movement time, main effects of visual field and object size, along with most interaction effects, were not significant (*F*'s ≤ 1.09, *p* ≥ 0.31). However, we did find a main effect of digit [*F*_(1, 20)_ = 54.07, *p* < 0.001, η^2^ = 0.16] and its interaction with visual field [*F*_(1, 20)_ = 7.41, *p* = 0.01, η^2^ = 0.003] such that the thumb showed faster movement times in the left VF whereas the index finger showed faster movement times in the right VF (Figure 7C). In general, the results here suggest that visual fields did not affect overall timing during grasping with one hand, although the index and thumb showed different visual field advantages.

#### Training effects

To look for learning effects, we calculated group averages based on the first and second half of grasping trials for each participant. Trends showed the same direction of visual field effects the first half of trials compared to the overall analyses above. However, in the second half of trials, visual field differences were greatly reduced, in particular for the slope and MGA (absolute and proportional).

#### Influences of left-hand proficiency

To look for possible influences of left-hand proficiency on our results, and importantly the unexpected visual field effect for MGA variability, we recalculated group averages for all the dependent variables based on participants who demonstrated good left-hand proficiency based on more accurate scaling of the grip to object size (i.e., minimum slope = 0.5). Although this reduced the reliability of our data, trends showed the same direction of effects in most cases, suggesting that the visual field effects on left-hand grasping cannot be explained by some form (or lack) of proficiency with using the left hand.

#### Factorial structure of grasp performance

To better understand the dimensions of grasp performance governing our participants' reach-to-grasp performance, we submitted all seven dependent variables for both the left and right VF (averaged across object width and/or digit where appropriate) to factor analyses, one for Experiment 1 and one for Experiment 2. Both factor analyses extracted 4 factors (eigenvalues > 0.5; varimax rotation, extraction method = principal component analysis). Table 1 provides a summary of the solution found for Experiment 1 with the loads of the different variables on the four factors (loads higher than 0.3 are bolded; loads smaller than 0.3 can be considered insignificant). The result suggests one factor of timing and three factors of grasp metrics (one primarily for MGA, one for standard deviation of MGA, and one primarily for slope). Table 2 provides a summary of the solution found for Experiment 2. The result confirms the separation of temporal and metric measures. However, there now are two factors of timing, one rather associated with reaction time (Factor 2) and the other more associated with movement time (Factor 3). Two additional factors explain variability of metric aspects of grasping: Factor 1 captures MGA (absolute and proportional) and slope, and Factor 4 captures standard deviation of MGA. Although more research is warranted, both factor analyses agree that the standard deviation of MGA loads onto a separate factor. Together with the non-intuitive results for left-hand grasping, this arguably indicates that standard deviation of MGA reveals processes that are separate from reach-to-grasp movement performance.

**Table 1:** **Right-hand grasping factor analysis rotated component matrix**.

**Dependent variable**	**Component**			
	**1**	**2**	**3**	**4**
**SLOPE**				
Left VF	−0.047	**−0.519**	0.162	**0.793**
Right VF	−0.196	**−0.357**	−0.140	**0.883**
**MGA**				
Left VF	0.110	**0.983**	0.038	−0.082
Right VF	0.134	**0.898**	−0.110	**−0.362**
**PROPORTIONAL MGA**				
Left VF	0.109	**0.979**	0.028	−0.123
Right VF	0.142	**0.880**	−0.094	**−0.409**
**STANDARD DEVIATION OF MGA**				
Left VF	0.174	−0.020	**0.945**	−0.151
Right VF	0.042	−0.062	**0.971**	0.149
**REACTION TIME**				
Left VF	**0.944**	0.082	−0.051	−0.060
Right VF	**0.887**	0.100	0.205	−0.066
**TIME MGA**				
Left VF	**0.943**	0.078	−0.033	−0.111
Right VF	**0.954**	0.070	0.139	−0.058
**MOVEMENT TIME**				
Left VF	**0.963**	0.134	−0.013	−0.105
Right VF	**0.940**	0.129	0.152	−0.016

*Loadings greater than 0.3 are highlighted in bold*.

**Table 2:** **Left-hand grasping factor analysis rotated component matrix**.

**Dependent variable**	**Component**			
	**1**	**2**	**3**	**4**
**SLOPE**				
Left VF	**−0.940**	−0.102	−0.039	−0.022
Right VF	**−0.841**	−0.199	−0.139	−0.237
**ABSOLUTE MGA**				
Left VF	**0.961**	−0.135	0.099	0.209
Right VF	**0.866**	**−0.381**	0.088	0.225
**PROPORTIONAL MGA**				
Left VF	**0.964**	−0.126	0.097	0.202
Right VF	**0.874**	**−0.363**	0.091	0.228
**STANDARD DEVIATION OF MGA**				
Left VF	**0.333**	0.102	0.175	**0.909**
Right VF	**0.513**	0.258	0.091	**0.730**
**REACTION TIME**				
Left VF	−0.143	**0.938**	0.070	0.040
Right VF	−0.129	**0.922**	0.157	0.049
**TIME MGA**				
Left VF	−0.037	**0.708**	**0.454**	0.285
Right VF	0.004	**0.798**	**0.460**	0.123
**MOVEMENT TIME**				
Left VF	0.202	0.177	**0.903**	0.174
Right VF	0.121	**0.385**	**0.852**	0.029

*Loadings greater than 0.3 are highlighted in bold*.

#### Omnibus analysis of visual field preferences

As the final step of our data analysis, we compared visual field preferences in the two experiments. A first approach used ANOVAs with mixed design (between-subjects factor: right-hand users vs. left-hand users; within-subject factor: contralateral vs. ipsilateral visual field; data were averaged across object width, and/or digit). To sort out any possible effects of handedness level between the two groups of participants, we conducted all ANOVAs with and without the participants' handedness laterality score as a covariate, but this had no influence on the pattern of significant *F*-tests. Also, to keep numbers of tests small, these analyses focused on three dependent variables that had produced significant visual field effects for each experiment separately: slope of MGA, absolute MGA and proportional MGA. Standard deviation of MGA was not considered given its apparently idiosyncratic underlying mechanisms. A complete account of the ANOVA results including handedness as a covariate is provided in Table 3. In summary, we observed a significant group-by-visual field interaction for slope (*p* = 0.026, η^2^_*p*_ = 0.103) indicating a more pronounced visual field difference for left hand grasping in terms of grasp-relevant object-size processing. Closer inspection of the right-hand data showed equal variability for slopes in the left and right VF. This rules out the possibility that the smaller visual field effect, compared to left-hand data, was caused by a ceiling effect. In addition, all three measures revealed a main effect of hand use, with poorer performance for left (non-dominant) hand use (slope: Left-Hand – Right-Hand = −0.5 mm; proportional MGA: Left-Hand – Right-Hand = 0.05; respectively; *p*'s < 0.041; absolute MGA: Left-Hand – Right-Hand = 2.59 mm, although this could be affected by placement of the markers). Moreover, and as expected, all three measures produced general visual field effects such that there was a visual field advantage for the side ipsilateral to the respective hand (*p*'s < 0.008).

**Table 3:** **Mixed-design ANOVA results**.

**Dependent variable**	**F1**	**F2**	**F1 × F2**
Slope	*F*_(1, 46)_ = 7.81	*F*_(1, 46)_ = 13.43	*F*_(1, 46)_ = 5.28
	*p* = 0.008^**^	*p* = 0.001^**^	*p* = 0.0260^*^
	η^2^_*p*_ = 0.145	η^2^_*p*_ = 0.226	η^2^_*p*_ = 0.103
Absolute MGA	*F*_(1, 46)_ = 11.41	*F*_(1, 46)_ = 4.41	*F*_(1, 46)_ = 0.055
	*p* = 0.001^**^	*p* = 0.041^*^	*p* = 0.815
	η^2^_*p*_ = 0.199	η^2^_*p*_ = 0.087	η^2^_*p*_ = 0.001
Proportional MGA	*F*_(1, 46)_ = 11.77	*F*_(1, 46)_ = 45.16	*F*_(1, 46)_ = 0.002
	*p* = 0.001^**^	*p* < 0.001^**^	*p* = 0.961
	η^2^_*p*_ = 0.204	η^2^_*p*_ = 0.495	η^2^_*p*_ < 0.001

*Within-subjects factor: contralateral vs. ipsilateral visual field (F1); Between-subjects factor: right-hand users vs. left-hand users (F2)*.

* *p < 0.05*,

** *p < 0.01*.

**Figure 8:**
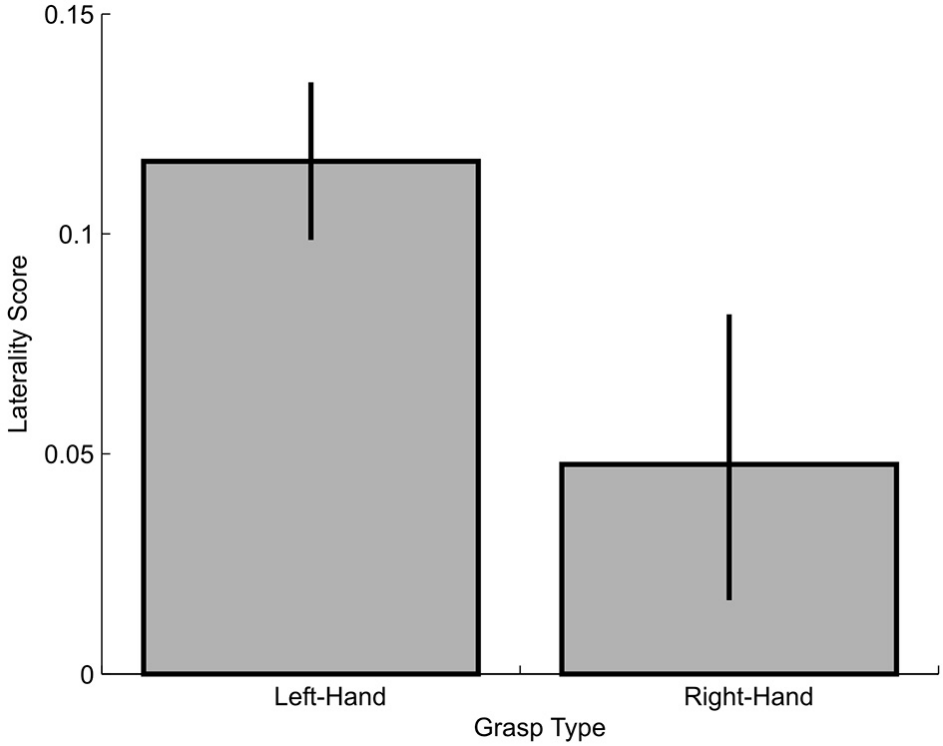
**Laterality score for left-hand and right-hand grasping**. Higher scores indicate more lateralized visual field effects.

In the second approach, we compared the overall size of the visual-field effect for left- vs. right-hand grasping by conducting a bootstrapping procedure. To do this, for each grasp-type, we calculated an averaged laterality index ([contralateral VF – ipsilateral VF]/[[contralateral VF + ipsilateral VF]/2]) across the three dependent variables that had produced significant visual field effects (slope, absolute and proportional MGA), such that the higher the index value, the greater the general visual-field effect. Then, we used random selection with replacement to re-create the groups of participants for the two experiments (*n* = 28 for Experiment 1, *n* = 21 for Experiment 2) and calculated group means of the laterality indices. Next we repeated this procedure 10,000 times to then determine percentiles of 50% for the medians as well as 2.5 and 97.5% for the confidence intervals (Figure 8). As shown, there we found that left-hand grasping had significantly greater overall visual-field effects than right-hand grasping. Thus, the results here confirm that left-hand grasping has a more pronounced preference for the ipsilateral visual field compared to right-hand grasping.

## Discussion

In the present study, we tested whether visual input of unimanual grasping varies as a function of visual field. Equivalent lateralizations are well known for the control processes of motor output for grasps with the right or left hand. That is, grasping with one or the other hand is controlled by the contralateral hemisphere (Rice et al., 2007) with a relative dominance of the left hemisphere for certain aspects of grasping (Davare et al., 2007). Consistent with our predictions, we found similar lateralizations for the visual input: for right-hand grasping, objects appearing in the right visual field (i.e., fixation to the left) were grasped with greater proficiency than when grasped in the left visual field (i.e., fixation to the right). In contrast, for left-hand grasping, objects in the left visual field were grasped with greater proficiency than when grasped in the right visual field. This suggests that sensorimotor demands for using the left- vs. right-hand trigger grasp circuits in the contralateral hemisphere, which is more sensitive to the respective contralateral visual field. Moreover, we found that visual field differences for left-hand grasping were more pronounced compared to the differences observed for right-hand grasping. In the following we will discuss contralateral specialization and differences between left- and right-hand grasping separately.

### Evidence for visual field lateralization

The finding of lateralized specialization is consistent with previous research. For bimanual grasping of a single object we have found visual field differences with a preference for the left visual field (Le and Niemeier, 2013a,b), which is the field contralateral to the hemisphere that controls bimanual grasps (Le et al., 2014). For observations of unimanual grasp actions, Shmuelof and Zohary (2006) found that brain activity in the right hemisphere (but not the left hemisphere, see below) varied as a function of visual field. Given the employed visual stimuli there is a good possibility that visual input for actual unimanual grasping might be treated similarly. Nevertheless, to our knowledge the present data are the first to directly demonstrate that visual object analysis used for overt unimanual grasping shows a preference for visual information from the visual field on the same side as the grasping hand.

We found this visual field advantage for our kinematic measures of MGA and MGA scaling. These measures have been demonstrated to reflect the proficiency of the grasp component for reach-to-grasp movements (Jeannerod, 1981, 1984; Tresilian and Stelmach, 1997). For example, for right-hand grasping in the right visual field our participants showed scaling of the MGA well within the normal ranges of proficient grasping (Smeets and Brenner, 1999). This said, direct comparability is limited because our participants grasped objects without directly looking at them, which should make grasping more challenging. Still, our participants grasped only two object widths so that they had a greater chance to memorize proper MGA sizes compared to grasp paradigms with multiple different object sizes. Nevertheless, right-hand MGA scaling in the left visual field was significantly reduced to a level at the lower range of functional grasps or below. For left-hand grasping we found the reversed pattern with worse scaling in the right than the left visual field (on overall lower levels of proficiency, see the section on left- vs. right-hand grasping).

In addition, we found that MGAs in the visual field ipsilateral to the grasping hand were smaller than in the contralateral visual field. This is consistent with observations that MGA increases with visual uncertainty, arguably in an attempt to “err” in a direction where the fingers are less likely to collide with the object (e.g., Schlicht and Schrater, 2007). Interestingly, the difference in MGA size was more apparent for the narrow object width as opposed to the wide object width. It is possible that the difference reflects a natural biomechanical constraint of opening up the distance between thumb and index finger beyond a certain point. However, this is unlikely given that we have observed a similar object size effect for bimanual grasping of similarly sized objects, so when there is no similar biomechanical constraint (Le and Niemeier, 2013a). Because larger MGAs occur at later stages of the reach-to-grasp movement (Jakobson and Goodale, 1991), together these data could be reconciled such that visual field effects might be more apparent for MGAs attained at earlier times of the movements. However, further research is required to confirm that smaller objects with earlier MGAs yield more pronounced visual field effects.

The present finding that visual field differences are reduced for wider objects (which have later MGAs) rules out the possibility that the visual field effects were caused by hand-distractions on one side of the object (e.g., right-hand grasps are more distracting for right fixations). That is, if the hand was a potential distraction, we should see stronger visual field effects for wider objects because the MGA would have been wider and closer to the object; however, we did not observe this. Moreover, it is unlikely that the hand trajectories would have produced distractions by passing the line of sight because hand movements started from a position below the object and fixation points. Even so, visual feedback of the hand generally does not have a facilitative effect (and hence distracting effect) on grip aperture during reach-to-grasp movements (Connolly and Goodale, 1999).

Unlike MGA and MGA scaling, MGA variability exhibited visual field differences that eluded any straight forward interpretation. For bimanual grasping, this measure was smaller in the dominant left visual field (Le and Niemeier, 2013a,b). Thus, smaller MGA variabilities seemed to serve as measures of greater grasp precision and, therefore, grasp proficiency. But, for unimanual grasping we found opposite trends: grasping with the left hand produced less MGA variability than grasping with the dominant right hand, and left-hand grasping in the (otherwise preferred) left visual field increased MGA variability. Also, the factorial structure of our dependent variables always had MGA variability load on its own factor, separate from other grasp metrics. So in sum, MGA variability is a measure that might be rather disconnected from other grasp measures and require further investigations of the underlying mechanisms.

More research will also be necessary to clarify why visual field presentation modulated only the metrics of the grasps, and not the timing or stability. One explanation could be task difficulty: Unimanually grasping an object that has a suitable width (i.e., as in the present study) may be easy and natural, thus allowing for an equal advantage for both visual fields in grasp timing and stability. In contrast, unimanual grasping an object that may be too wide or narrow might require increased inter-digit coordination in both metrics and timing, due to the increased level of difficulty in keeping the grasp stable, and thereby increasing visual field effects. Consistent with this explanation, we found visual field effects for the timing as well as metrics in our previous visual field study on bimanual grasping of small objects (Le and Niemeier, 2013a), which are presumably more difficult compared to bimanual grasping of larger objects. Indeed, when we examined bimanual grasping of large objects, we found visual field effects for the metrics only (Le and Niemeier, 2013b).

### Differences between left- and right-hand grasping

Left- and right-hand grasping differed in several ways. As expected, non-dominant left-hand grasping showed signs of reduced proficiency: MGA was larger for left-hand grasping and MGA scaling showed flatter slopes than right-hand grasping. Our skewness data suggested that no MGA ceiling effects during left hand grasping and thus the comparatively flat slopes (Smeets and Brenner, 1999) were unlikely caused by ceiling effects during left-hand grasping, such as, limits of hand span that would limit MGA sizes of the wider object width. If at all, MGA showed limitations during right-hand grasping. Nevertheless, its slopes were steeper—and thereby closer to ideal—than usual (Smeets and Brenner, 1999). At any rate, limitations imposed by hand span could not explain visual field specific differences. It is possible however that, as one contributing factor, grasp performance in the two experiments differed because different participants were tested. Importantly, people in the second experiment showed higher handedness scores (Oldfield, 1971). Although the handedness inventory is a relative measure it could reflect that Experiment 2 participants were systematically less proficient with their left hand. However, if at all, the handedness difference seems to have played a small role because controlling for the influence of handedness did not alter the results in the omnibus ANOVAs.

Interestingly, left- and right-hand grasping differed in the strength of the visual field effects; specifically, left-hand grasping showed substantial differences in MGA scaling in the left and right visual field but for right-hand grasping the differences were less pronounced so that the omnibus ANOVA flagged a significant group-by-visual field interaction. The interaction was strong enough to generate a greater bootstrapped lateralization score for left- than right-hand grasping. Once again, it is possible that unspecific differences between participant groups contributed to the interaction. However, these differences might have contributed relatively little because handedness as a covariate was unsuccessful in explaining the group-by-visual field interaction. Moreover, only MGA scaling showed an interaction but not the other MGA measures, whereas an unspecific group differences should have had a generalized influence on interactions for multiple measures.

We speculate that the difference in visual field effects could reflect two possible causes. First, people could have scaled right-hand grasps better and less differently for the visual fields because right-hand grasping has more privileged visuomotor coupling or is better capable of forming proprioceptive or procedural memories of grasps in the course of the experiment. Especially with two object sizes only, visual field differences could have leveled out for right-hand grasping, although here we found little evidence for training effects in Experiment 1. Still, left-hand grasping might have relied more on online control (e.g., Haaland and Harrington, 1989; Haaland et al., 2004; Gonzalez et al., 2008; Tremblay et al., 2013). If so, it still remains unclear why MGA scaling showed a group-by-visual field interaction but not the MGA measures. Moreover, we found that the visual field differences leveled out by the second-half of the trials for left-hand grasping instead. Thus, a second perhaps more plausible cause of the interaction is that the left hemisphere is better equipped to perform visual object analysis across the two visual fields. For example, left-hemisphere areas performing visual object analysis for grasping, likely including aIPS (Rizzolatti and Luppino, 2001), might contain neurons with visual receptive fields that expand further into the left visual field compared to equivalent areas in the right hemisphere. This interpretation agrees well with Shmuelof and Zohary's (2006) observation that activity in right-brain grasp areas varied as a function of visual field but not activity in the left hemisphere. Consistent with this idea, that sensorimotor control in the left hemisphere might have a greater need to access visual information in both visual fields to permit greater flexibility in grasp actions of the dominant right hand across the entire visual field.

In conclusion, here we investigated visual field effects on unimanual grasping. We found a visual field advantage for the side ipsilateral to the grasping hand indicating a contralateral organization of visual object analysis. In addition, we observed differences in the degree of lateralization that could reflect a relative dominance of the left hemisphere for the visual analysis component of prehension. Our data contribute to the mechanistic understanding of the visual processes that give rise to the sensorimotor control of grasping. In addition, our findings might be of clinical significance as they could help refine rehabilitation programs for patients with motor deficits after cortical damage such as stroke (e.g., Metrot et al., 2012).

### Conflict of interest statement

The authors declare that the research was conducted in the absence of any commercial or financial relationships that could be construed as a potential conflict of interest.
